# Analysis of clinical and genetic features among 12 neonatal diabetes mellitus

**DOI:** 10.1186/1687-9856-2013-S1-P5

**Published:** 2013-10-03

**Authors:** Cao Bingyan, Gong Chunxiu, Yang Jinkui, Cao Xi, Wu Di

**Affiliations:** 1 Beijing Children’s Hospital, Capital Medical University, Beijing, China

## Objectives

To analyze the clinical and genetic features among 12 neonatal diabetes mellitus (NDM).

## Methods

Describe clinical features of 12 diagnosed NDM cases in my hospital between Jul. 2001 and Jun. 2012. KCNJ11, ABCC8 and INS gene were sequenced in all patients.

## Results

The age at diagnosis was between 0.5 and 5 month, the median was 3 month. 7 cases (58.3%) were SGA. Infection occurred in 7 cases, 4 cases with convulsion and 8 cases with ketoacidosis. The mean HbA1c at diagnosis was 10.0% (7.4%~13.7%). Insulin treatment was started in all 12 patients, the initial dose was 1.0~1.2IU/kg/d, 6 cases were treated with glyburide after the acute phase, only one boy who diagnosed as DEND syndrome reached euglycemia. 2 cases stopped glyburide because of gastrointestinal adverse reaction. Among the 12 cases followed up, 7 had PNDM, 4 had TNDM, 1 case lost following, 2 cases died, one was the DEND syndrome patient died of DKA, the other died of hepatic and renal failure at the age of 1 year and 6 months. One had skeletal dysplasia and diagnosed as Wolcott-Rallison syndrome. The blood glucose of most patients was well controlled. KCNJ11c.175G>A (V59M) and KCNJ11c.601C>A (R201H) mutation were found in two patients.

## Conclusion

The clinical expressions of NDM were atypical and easily missed diagnosis. Of all the NDM cases, 30% was TNDM and relieved automatically. Insulin was the best choice before genetic identification. The KCNJ11 mutation rate of PNDM was 28% in China.

